# Valacyclovir-Induced Thrombotic Thrombocytopenic Purpura

**DOI:** 10.7759/cureus.8156

**Published:** 2020-05-16

**Authors:** Sumera Bukhari, Hafiz M Aslam, Talha A Awwal, Donald Christmas, Sara L Wallach

**Affiliations:** 1 Internal Medicine/ Hospital Medicine/ Palliative Medicine, Cambridge Health Alliance-Harvard Medical School, Cambridge, USA; 2 Internal Medicine, Hackensack Meridian School of Medicine at Seton Hall University, Nutley, USA; 3 Internal Medicine, St. Francis Medical Center, Seton Hall University-Hackensack Meridian School of Medicine, Trenton, USA

**Keywords:** ttp, thrombotic thrombocytopenic thrombocytopenia, valacyclovir

## Abstract

Valacyclovir is a well-tolerated antiviral drug. Thrombotic thrombocytopenic purpura is a rare adverse effect of valacyclovir therapy. Mostly, it has been reported in clinical trials and case reports in patients with high dose or low dose therapy in immunocompromised patients. Herein we write a case report of the immunocompetent patient, who was taking very low dose valacyclovir therapy for his recurrent genital herpes. This case emphasizes the role of low dose (1000 mg/day) valacyclovir therapy causing thrombotic thrombocytopenic purpura in an immunocompetent patient with no other explainable trigger.

## Introduction

Valacyclovir is an antiviral prodrug of acyclovir. It is active against herpes simplex virus types 1 and 2, varicella-zoster virus, Epstein-Barr virus, and on a high dose, it has also shown to be effective against cytomegalovirus [1]. Valacyclovir is a well-tolerated drug with few adverse effects of headache, nausea, and abdominal pain. However, acute renal failure and central nervous system adverse reactions have been reported in elderly patients with underlying kidney disease. Thrombotic thrombocytopenic purpura (TTP) has occurred in patients with advanced HIV disease and allogeneic bone marrow transplant and renal transplant patients receiving 8 grams per day of valacyclovir in clinical trials [2,3]. To the best of our knowledge, one case report of TTP at a low dose of valacyclovir therapy has been reported in the immunocompromised patients [4]. Herein we write a case of valacyclovir-induced thrombotic thrombocytopenic purpura on a very low dosage (1000 mg/day) in an immunocompetent patient.

## Case presentation

A 37-year-old male with a history of recurrent genital herpes taking valacyclovir for a year presented with progressive shortness of breath on exertion with palpitations and blood in the urine for one week. The patient has no other medical condition and is taking no other medication like quinine or anti-platelets. He denies drinking alcohol or smoking, except using marijuana occasionally. Family history is non-contributory with no blood disorders. On admission, the patient was awake, alert, and oriented with no acute distress. His temperature was 98.7 °F, blood pressure 136/86 mmHg, and pulse 120 beats per minute. He had no neurological sensory or motor deficit. Except for sinus tachycardia, the rest of the physical exam was also unremarkable.

Laboratory data (Table 1) was remarkable for severe hemolytic anemia and thrombocytopenia. Peripheral smear revealed numerous schistocytes. The renal and liver function tests were normal except elevated indirect bilirubin. Urinalysis showed the presence of proteins and many erythrocytes. His coagulation profile was normal. The chest X-ray was clear. The electrocardiogram showed sinus tachycardia. The patient was admitted to the intensive care unit with severe hemolytic anemia and thrombocytopenia secondary to thrombotic thrombocytopenic purpura. ADAMTS-13 activity levels were severely low. Human immunodeficiency virus (HIV) and the direct antiglobulin (coombs) tests were negative. Vasculitis and autoimmune panel was negative on screen. Computerized tomography scan of the brain and echocardiogram were unremarkable. Leukocytosis was likely reactive and secondary to steroids use. Blood and urine cultures did not grow any organism. Cytomegalovirus and Babesia titers were negative. Valacyclovir was discontinued on admission, and he received emergent plasmapheresis in first 24 hours and high dose steroids. His symptoms improved considerably with a substantial rise of platelets and hemoglobin on subsequent plasmapheresis sessions in next 48 hours. His hematological parameters became normal in 3-4 days, and his symptoms resolved at the time of discharge. He remained in remission on follow-up after one month of hospital discharge.

**Table 1:** Laboratory Values

Laboratory Values			
Name of Test	On Admission day	On Discharge	Reference range
Hemoglobin	6-5	11.8	14-18 g/dl
Hematocrit	19.1	37.6	42-52%
Platelets	8	199	130-400 /mL
White Blood Cells	16.5	10.2	4.8-10.8 /mL
Retic Count	10.2	5.8	0.5 – 1.6%
Lactate dehydrogenase	2810	188	125-225 U/L
Serum Bilirubin (Direct/Total)	3.6/0.5	0.4/0.2	0.1-1/0.1-0.3 mg/dL
Urea Nitrogen	22	18	6-20 mg/dL
Creatinine	0.92	0.98	0.7-1.2 mg/dL

## Discussion

Thrombotic thrombocytopenic purpura (TTP) is a rare, life-threatening disorder of the blood coagulation system, causing extensive microscopic clots to form in the small blood vessels throughout the body. The classic pentad of TTP includes thrombocytopenia, microangiopathic hemolytic anemia, fever, neurological findings, and kidney function abnormalities [5]. However, only thrombocytopenia and microangiopathic hemolytic anemia without an apparent alternative cause is required to diagnose TTP [6]. Moderate to severe deficiency (activity <10%) of ADAMTS13 (a metalloprotease to cleave von Willebrand Factor multimers) appears to be associated with many cases of TTP [7]. TTP can be hereditary, due to inherited mutations in ADAMTS13 or acquired, due to an autoantibody inhibitor to ADAMTS13. Conditions and factors associated with acquired TTP are numerous and include most notably infections, but also autoimmune disorders, malignancy, and a variety of drugs [8].

Drug-induced TTP accounts for ~15% of TTP cases [8]. The majority of drug-induced TTP case reports are challenging to interpret because there is ambiguity regarding the relation of drug exposure to the onset of TTP [9]. Medina et al. studied various drugs causing TTP and postulated that the mechanisms of drug-induced TTP might be either immune-mediated (autoantibodies to ADAMTS13) or direct toxicity (dose-related). The most common drugs causing TTP are mitomycin C and cyclosporine (dose-related toxicity), quinine, ticlopidine, and clopidogrel (immune-mediated reaction) [9].

The exact mechanism of TTP by valacyclovir remains unclear. However, it can be explained by the decreased activity of ADAMTS13 noticed in this case, suggesting an immune-mediated mechanism. The patient had severely reduced ADAMTS13 activity <3% (Normal Lab range >68%) initially, and the activity increased to 98% during the remission phase. The patient showed the same characteristic manifestations of TTP as it was noticed in the cytomegalovirus prophylaxis trial on high-dose valacyclovir [3]. There was no associated infection, and the onset of TTP was gradual as it occurred after 6-12 months of prolonged therapy. The delayed onset is contrary to the explanations of most immune-mediated TTP, which are often acute. Dose-related direct drug toxicity is often delayed [9,10].

## Conclusions

TTP is almost always fatal if appropriate treatment is not initiated promptly. The drug-induced TTP is managed effectively by the discontinuation of the offending drug and starting the plasma exchange. The response to the treatment of TTP after starting the plasma exchange is usually observed in the first three weeks in about 80-90% of cases. If there is no response to plasmapheresis and steroid treatment, other treatment options, including intravenous immunoglobulin, cyclophosphamide, vincristine, and rituximab, should be considered. It’s essential to recognize a drug-associated etiology for TTP, to avoid re-exposure and recurrent life-threatening illness with variable prognosis.
